# Letter to the Editor GHFBB; Response to Peña AS: What is the best histopathological classification for celiac disease? Does it matter?

**Published:** 2016

**Authors:** Olga M Pulido

**Affiliations:** *Department of Pathology and Laboratory Medicine, University of Ottawa, Ottawa, Ontario, Canada*

**To The Editor:**

The editorial by Peña AS: (1) and the responses elicited by it are of major importance. Everyone concur that patient’s wellbeing; proper diagnosis and treatment are the main objective. Let’s not forget that these concepts and guidelines are used by those rendering the direct health service to the public, by policy makers and regulators, which often need to relay on the opinion of experts on the field. Hence, communication and transfer of information needs to be clear, and applicable to patient care and public health. Evaluation of new therapeutic products to manage celiac disease (CD), requires agreement on guidelines for diagnosis (Dx) and follow up, including: diagnostic methods, reproducibility, validation and sensitivity. Standardization of best practice guideline may improve detection of CD in regions with limited human resources and equipment. Recruiting of CD patients for clinical trials is usually challenging, particularly because the need of biopsy as part of the Dx. The lack of animal models to assess new therapeutic compounds enhances the need to use clinical trials at earlier stages of drug development, adding a challenge and need for standardization of Dx guidelines. With these in mind: 

I agree that in cases of suspected CD and conditions linked to adverse reactions to gluten, small bowel biopsy procedures, including: sampling, processing, evaluation tools, classification and interpretation are key for differential diagnosis and management. The need of standardizing histopathology classification that can be used worldwide is essential. Peña AS (1), provide a very useful tabulated comparison among different classifications, facilitating the interpretation of publications using various classifications, allowing for compilation and analysis of data for public health. The idea of re-assessing the emphasis on the biopsy as a gold standard in the diagnosis of CD, in light of available less invasive tests, is a welcoming one, at least reducing the number of biopsies required for follow up. Villanacci (2) emphasizes on reproducibility and the use of simpler classification to facilitate reproducibility, but his suggested classification seems over simplified. Clinicians shall decide if that will be sufficient for proper Dx and management. Villanacci (2, 3) points out the advantage of including the term of “Microscopic Enteritis” (4) as a separate histopathology Dx, a practical suggestion for differential diagnosis. Marsh, Villanacci and, Srivastava (5) illustrate the differences between the histopathology of CD Marsh classification (4), and non-celiac gluten sensitivity. Despite its limited explanation, this publication is very useful to pathologists. Could this classification be modified not to use morphometric analysis? Do the Villanaci (2, 3) simplified classification provide the information required for best practice? These questions need consensual response for best practice guidelines worldwide. Nonetheless and until proven otherwise evaluation of new therapeutic products are best to use the Marsh classification (5). It calls my attention that in this discussion there is no mention of differences in diagnostic approach between pediatric and adult cases, since the need for the use of biopsy in pediatric has been questioned. Do you have any thoughts on that?

The concern expressed by Walker (6) is a valid one, but if anything is complementary to Peña AS (1) editorial. International guidelines cited are the key to standardization on diagnosis and management of CD, and gluten free-diet (GFD) as the therapy of choice, which carries an emotional, and financial impact. The ACG Clinical Guidelines: Diagnosis and Management of CD published by Rubio-Tapia et al. (6) indicates that a diagnosis of CD requires the demonstration of histological changes associated with the disease, classified according to: Marsh, Marsh modified (Oberhuber), or by the more recent, simplified Corazza classification. It accepts the incorporation of simplified classifications for the general practice.
